# Association Between Hair Trace Element Content and Children’s Growth and Development: Protocol for a Cross-Sectional Surveillance Study

**DOI:** 10.2196/72207

**Published:** 2025-09-16

**Authors:** Gulnara Batyrova, Gulmira Umarova, Yeskendir Umarov, Gulaim Taskozhina, Victoria Kononets, Rabbil Batyrov

**Affiliations:** 1 Department of Clinical Laboratory Diagnostics West Kazakhstan Marat Ospanov Medical University Aktobe Kazakhstan; 2 Department of Evidence-Based Medicine and Scientific Management West Kazakhstan Marat Ospanov Medical University Aktobe Kazakhstan; 3 Department of Natural Sciences West Kazakhstan Marat Ospanov Medical University Aktobe Kazakhstan

**Keywords:** trace elements, child health, child development and growth, children, Kazakhstan

## Abstract

**Background:**

Western Kazakhstan, a major industrial region, faces environmental challenges from the release of toxic elements due to intensive industrial activities. The combined impact of anthropogenic factors, trace element deficiencies, and harsh climatic conditions contributes to the deterioration of child health and growth.

**Objective:**

This study aimed to investigate the elemental profile of the child population in the western region of Kazakhstan.

**Methods:**

A total of 2279 school-aged children (aged 5 to 18 years) who are permanent residents of the Aktobe, Mangystau, Atyrau, and West Kazakhstan regions will be included in the study using a cluster sampling method. The elemental composition of their hair will be analyzed using inductively coupled plasma mass spectrometry on an Agilent 8900 inductively coupled plasma mass spectrometer (Agilent Technologies). Children’s physical growth will be assessed according to the World Health Organization (2007) growth standards and characterized using the following *z* score values: body weight for age for children aged younger than 10 years, height for age, and BMI for age. The cutoff points of *z* score values make it possible to diagnose thinness, stunting, overweight, or obesity. Multiple linear regression analysis will be applied to assess the association between chemical element content in hair and *z* score measures of children’s physical growth, as well as associations with gender, age, sociodemographic factors, and residential status. Reference values for chemical element content in biological substrates of the western Kazakhstan population will be established using 95% coverage intervals with 95% CIs following International Union of Pure and Applied Chemistry recommendations. The chemical element content will be reflected in a web-based atlas.

**Results:**

After recruitment of participants, data will be collected between September 2023 and March 2025. Data processing and analysis will be completed in September 2025. Publication of the results is expected in December 2025. An analysis will be conducted to determine the differences in the levels of elements in groups of boys and girls, urban and rural children, and groups of children of different ages. According to the results of multiple regression analysis, chemical elements influencing the indicators of the physical development of children will be identified.

**Conclusions:**

The identified associations between trace and macroelement content and children’s growth indicators will allow for the development of region-specific public health measures, such as nutritional supplements, environmental remediation, and policies aimed at reducing exposure to toxic elements. In addition, identifying differences between rural and urban populations could inform the development of targeted prevention strategies. The developed web-based atlas of trace and macroelement content will be necessary for further research on the prevalence, etiology, risk factors, and possible mechanisms of development of environmentally dependent, endemic diseases in the region. Reference values of trace and macroelement content will also be established.

**International Registered Report Identifier (IRRID):**

DERR1-10.2196/72207

## Introduction

### Background

The physical growth of children is defined as the irreversible and permanent increase in size, whereas development refers to the enhancement of psychomotor abilities. Both processes are largely influenced by genetic, nutritional, and environmental factors. Physical development is also shaped by socioeconomic conditions, family characteristics, malnutrition, and micronutrient deficiencies [1].

For optimal physical growth and development in children, adequate intake of trace and macroelements is essential. Insufficient intake of essential and conditionally essential elements can disrupt normal physical growth and mental development in children [2]. For instance, selenium plays a critical role in protecting cells from oxidative stress, maintaining resistance to infectious agents, and regulating tissue growth and differentiation processes [3].

Zinc is involved in key biological processes, including cell growth and differentiation, gene expression, and protein synthesis. Zinc deficiency can lead to growth retardation, impaired innate and adaptive immunity, and disrupted regeneration processes [4]. Moreover, zinc is closely associated with indicators of physical development and growth in children [5].

Iodine is an essential microelement required for the production of thyroid hormones. Iodine deficiency can result in goiter, hypothyroidism, miscarriage in pregnant women, stillbirth, congenital anomalies, infant and neonatal mortality, and growth failure. Adequate thyroid hormone levels are crucial for normal growth and the development of the nervous system during fetal development, infancy, and childhood [6].

In contrast, toxic metals, as environmental factors, negatively affect the physical development of children. Research indicates that arsenic, cadmium, and lead adversely influence growth and development at various ages. While lead has been shown to negatively impact cognitive development in children, the effects of mercury on physical development remain less understood [7].

Western Kazakhstan is the largest industrial region in the country. The operation of metallurgical, chemical, and oil industry enterprises, as well as the extraction of oil shale, potassium-magnesium salts, limestone, cement raw materials, chalk, expanded clay, and construction sand, results in the release of toxic chemical elements into the environment. This region is classified as a boron and chromium biogeochemical province, and iodine deficiency is endemic.

The combined effects of anthropogenic factors, deficiencies in vital trace elements, and the unfavorable climatic and geographical conditions in which much of the population of Western Kazakhstan resides contribute to a decline in health at both the individual and population levels [8-10].

The tense ecological situation on the territory of western Kazakhstan contributes to shifts in the trace element balance of the biosphere. The bioelement background of the living environment is reflected in the bioelement status of the human organism, leading to the deterioration of public health. Children are highly vulnerable to environmental pollutants, including toxic trace elements, which is why they are a key focus of environmental and public health research [11,12]. They are more negatively affected by toxic elements due to their behavioral patterns: they live and play closer to the ground, and they put their hands and objects in their mouths [13,14]. They consume more food, water, and air per unit of body weight than adults and have a higher absorption rate from the gastrointestinal tract [15,16]. Toxic and potentially toxic elements negatively impact children’s development and health [17]. In addition, given the antagonistic and synergistic interactions between elements, excessive intake of essential trace and macroelements can also have negative consequences [17,18].

Recently, the number of studies assessing the elemental profile of the pediatric population has increased in various regions of the world, including Europe [11,12,18-26], Asia [27-34], North America [35-37], South America [38-40], Africa [17,41,42], and Australia and Oceania [43,44]. Many of these studies have found a link between the characteristics of the elemental profile and various factors, including the geochemical characteristics of the area of residence (eg, natural and anthropogenic contamination of areas with certain chemical elements) [22,45-47], diet [22,45,48-51], drinking water consumption [11,17,49], lifestyle [30,42,52,53], gender [17,21-25,38,54,55], age [22,54], and social differences [42,52]. Some studies focus on the relationship between the chemical element content in biological samples and children’s health indicators [45,48,56,57].

Our study is the first large-scale study of the elemental profile of the child population in Kazakhstan. Data on the chemical element content of the adult population are presented in our previous work [58,59]. However, reference intervals have not been calculated for the child population. Studying the relationship between element content and growth indicators will enable the identification of the impact of the environment and nutrition on children’s health in the region, which is necessary for effective risk assessment, early detection of micronutrient imbalances, and the development of measures to improve children’s health in western Kazakhstan. Creating a digital atlas accessible to all that reflects the trace element content will enable early prenosological diagnostics of diseases and the adoption of preventive measures to eliminate elemental imbalances in western Kazakhstan.

Our scientific hypothesis is that elemental profile is associated with indicators of physical growth in the child population of the western region of Kazakhstan. The chemical element content in the biosubstrates of the child population differs between rural and urban residents.

### Objectives

Our aim is to investigate the elemental profile of the school-aged children population living in the industrial western region of Kazakhstan.

The objectives of this study are as follows: (1) to assess the elemental profile of children in rural and urban areas, identify regional characteristics, and establish reference values for the chemical element content in the hair of children considering age and gender; (2) to evaluate the relationship between the chemical element content in hair and the physical growth and development of children in western Kazakhstan; (3) to develop maps illustrating the elemental profile of the child population in the western region of Kazakhstan; (4) to compare the elemental profile of children with that of the adult population to analyze ontogenetic changes in the concentration of chemical elements in the hair of western region residents; and (5) to compare the results of chemical element analysis in the hair of children from the western region with previously published data on other populations.

## Methods

### Research Design

**Figure 1:**
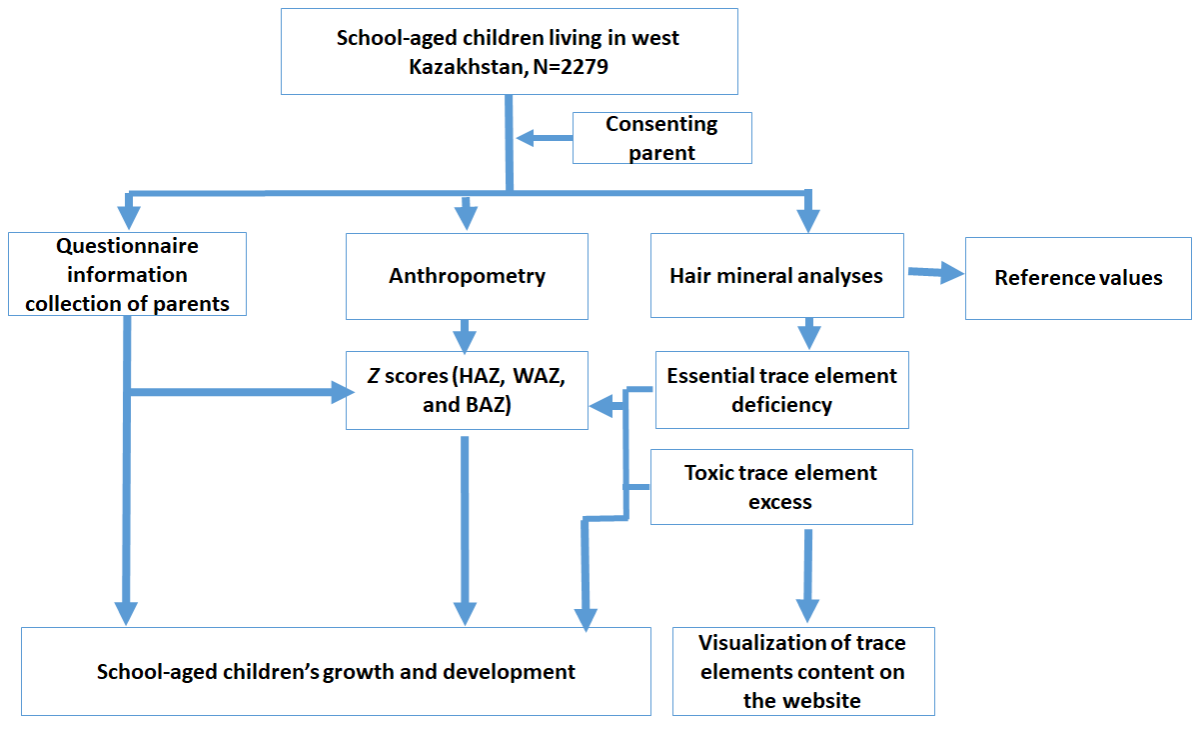
Flowchart of the study. BAZ: body mass index for age *z* score; HAZ: height for age *z* score; WAZ: weight for age *z* score.

This is a 1-stage, cross-sectional descriptive study (Figure 1). It will be conducted in populated areas of the western region of Kazakhstan: the Aktobe, Mangystau, West Kazakhstan, and Atyrau regions.

Inclusion criteria are as follows: (1) school-aged children (aged 5 to 18 years) who are permanent residents of the western region of Kazakhstan and (2) for whom written informed consent from parents or legal guardians is obtained before participation in the study.

Exclusion criteria are as follows: (1) comorbid somatic diseases, (2) endocrine or neurological disorders, (3) hereditary diseases and congenital developmental anomalies, and (4) use of vitamin or mineral supplements.

A total of 2279 school-aged children (aged 5 to 18 years) who are permanent residents of the Aktobe, Mangystau, Atyrau, and West Kazakhstan regions will be included in the study using a cluster sampling method. Schools will be randomly selected within each cluster, and within these schools, classes will be randomly chosen to form the study groups.

The sample size was calculated using official demographic data on the population aged 5 to 18 years across the 4 regions of western Kazakhstan [60]. The following formula was used to calculate the sample size:



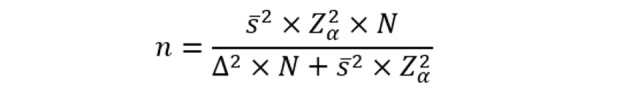



Where the average intragroup variance is:



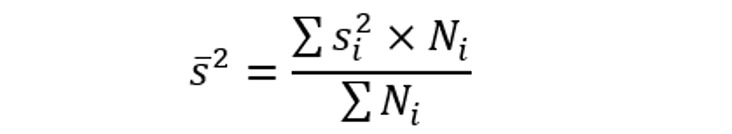



N*_i_* is the number of objects in each class of the general population (N_1_=204,124 in the Aktobe region; N_2_=195,572 in the Mangystau region; N_3_=168,154 in the Atyrau region; N_4_=136,255 in the West Kazakhstan region; N=704,105). Data from previous studies conducted in western Kazakhstan [9] were used for the calculation. With a Δ (sampling error) value of 0.04 and an α of .05 (*Z*=1.96), the total sample size was 1847. To account for possible data loss, 2279 children will be recruited for the study. The sample will be divided into 4 age groups: preschoolers aged 5 to 6 years, schoolgirls and schoolboys aged 7 to 10 years and 11 to 15 years, and adolescents aged 16 to 18 years [61].

### Study of the Elemental Profile of Children

Hair samples will be obtained using clean, stainless steel scissors from 3 to 5 areas of the occipital part of the head in quantities of at least 0.1 g. For elemental analysis of hair, proximal parts of strands of 3 to 4 cm in length will be used. Samples will be placed in envelopes with identification records.

The hair samples will be analyzed for the following 25 chemical elements: aluminum, arsenic, boron, beryllium, calcium, cadmium, cobalt, chromium, copper, iron, iodine, potassium, lithium, manganese, magnesium, sodium, nickel, phosphorus, lead, selenium, silicon, tin, vanadium, mercury, and zinc. The elemental composition of the hair will be analyzed using inductively coupled plasma mass spectrometry (ICP-MS) on an Agilent 8900 mass spectrometer (Agilent Technologies) using an SPS 4 automatic dispenser (Agilent Technologies; Table 1).

Hair samples will be subjected to sample preparation through washing and microwave decomposition. The hair strands will be washed using acetone and then rinsed 3 times with deionized water and air dried at 60 °C. The sample (sample weight=0.05 g) will be decomposed using nitric acid (Fluka 02650; Sigma-Aldrich) in a Berghof SW-4 DAP-40 microwave system (Berghof Products + Instruments GmbH) and diluted using distilled deionized water to a concentration of 0.5% to 1% of nitric acid before direct injection into the ICP-MS system.

The ICP-MS system is prepared for operation according to factory specifications and calibrated via external calibration against multielement standards. A tuning solution of cerium, cobalt, lithium, thallium, and yttrium of 10 μg/L in 2% acid (Agilent Technologies) is used for daily evaluation of the sensitivity of the mass spectrometer. The content of oxides and doubly charged ions does not exceed 1.5%.

Standards containing all elements to be assessed at concentrations of 0.5, 1, 5, 10, and 5000 μg/L are prepared before starting work using the Universal Data Acquisition Standards Kit reference solutions (PerkinElmer) through dilution in distilled deionized water acidified with 1% of nitric acid. To account for incomplete matching of sample matrices and calibration solutions for acidity and viscosity, the assay uses online internal standardization for rhodium isotope 103. An internal standard containing 10 μg/L of rhodium is prepared from a rhodium reference standard (PerkinElmer) on a matrix containing 8% 1-butanol (Merck KGaA), 0.8% Triton X-100 (Sigma-Aldrich), 0.02% tetramethylammonium hydroxide (Alfa Aesar), and 0.02% ethylenediaminetetraacetic acid (Sigma-Aldrich).

Sample collection is conducted using an SPS 4 autosampler (Agilent Technologies), and sample introduction is conducted using an Integrated Sample Introduction System 3 (Agilent Technologies). To eliminate polyatomic overlays, an ammonia-helium mixture is used as the reaction cell gas.

**Table 1:** Conditions of analysis.

Condition	Parameters
RF^a^ power	1550 W
Cooling gas flow	15 L/min
Auxiliary gas flow	0.9 L/min
Nebulizer gas flow	1.05 L/min
Sample introduction system	ISIS^b^ 3 for the Agilent 8900 mass spectrometer (Agilent Technologies) MicroMist nebulizer (standard for base model 100), and sample uptake rate of 0.4 mL/min (Agilent Technologies) Quartz double-pass Scott spray chamber (quartz spray chamber for the ICP-MS^c^ mass spectrometer part number G8400-67150; Agilent Technologies)
Reaction or collision cell gas	Ammonia and helium mixture
Reaction or collision cell gas flow	1 L/min
Cone material	Platinum
Injector	Fassel heater, single-piece quartz torch with a 2.5-mm internal diameter injector (standard for the Agilent 8900 ICP-MS mass spectrometer part number G3280-80053; Agilent Technologies)
Sample flow	400 μL/min
Internal standard flow	40 μL/min
Modes	No gas mode: analytes include lithium, beryllium, boron, aluminum, silicon, phosphorus, sulfur, nickel, copper, zinc, gallium, germanium, arsenic, bromine, rubidium, strontium, zirconium, niobium, molybdenum, palladium, silver, cadmium, indium, tin, antimony, tellurium, iodine, cesium, barium, lanthanum, tungsten, platinum, gold, mercury, thallium, lead, bismuth, and uranium Reaction or collision cell mode: analytes include sodium, magnesium, potassium, calcium, titanium, vanadium, chromium, manganese, iron, cobalt, selenium, and krypton
Dwell time per mass and scanning mode	Single quadrupole mode: 50-100 ms MS/MS^d^ mode: 100-200 ms
Wait time	Single quadrupole mode: 0 ms MS/MS mode: 7 ms
Peak profile mode	1 scanning point for the first quadrupole and 3 scanning points for the second quadrupole
Readings per replicate	3
Replicate number	3

^a^RF: radio frequency

^b^ISIS: Integrated Sample Introduction System.

^c^ICP-MS: inductively coupled plasma mass spectrometry.

^d^MS/MS: tandem mass spectrometry.

### Establishment of Reference Values of the Chemical Element Content

Establishment of reference values of the chemical element content in biological substrates of the population of the western region of Kazakhstan will be carried out by determining 95% coverage intervals with 95% CIs. The analysis will be carried out in accordance with the recommendations of the International Union of Pure and Applied Chemistry [62].

### Assessment of Physical Growth

Anthropometric parameters (body weight and height) of children will be assessed once. Measurement of children’s body weight will be carried out in electronic medical scales with an accuracy of 50 g. Height will be measured using a medical height gauge with a folding stool in the standing position. According to the manufacturer of the height gauge, the accuracy of the measurement is 0.1 cm.

Children’s physical growth will be assessed according to the World Health Organization (WHO; 2007) growth standards using the *z* score: the number of SDs (SD score; SDS) by which the anthropometric value differs from the median value of the standard population. A specialized program developed by the WHO, AnthroPlus, will be used to assess the growth of children [63].

When calculating *z* scores for children of the study sample, it is suggested to assume that they are equal to 0 for the standard population. Thus, the amount of deviation of sample *z* score values from 0 indicates the deviation of the growth indicators of children in this group from those of the standard population. To assess the physical growth of children and the influence of bioelements on it, we will use the method of anthropometric data assessment via *z* score, which consists of calculating the number of SDs or sigmas by which the studied indicator differs from the median of the standard population. If the anthropometric data of a particular child are lower than the median of the standard for a given age, the *z* score will be negative. Conversely, if the body mass or body length of a given child is above the median of the standard population, then the *z* score will have a positive value. Children’s anthropometric data are characterized using *z* score values: body weight for age for children aged younger than 10 years (weight for age *z* score; WAZ), height for age (height for age *z* score; HAZ), and BMI for age *z* score (BAZ). The cutoff points of *z* score values make it possible to diagnose thinness, stunting, overweight, or obesity. The use of *z* score calculation methodology to estimate the anthropometric parameters of children in epidemiological studies has certain advantages over other anthropometric indexes, in particular over the analysis of percentile distribution due to the possibility of statistical analysis and comparison of groups [64,65].

To study the relationship between element content and physical growth, we considered it appropriate to use this method.

The following indicators will be calculated: WAZ for children aged younger than 10 years, HAZ, and BAZ. According to WHO recommendations, the interpretation of the obtained *z* score values is as follows:

- WAZ: SDS of <−2 for thinness, SDS of –2 to +2 for normal values, and SDS of >+2 for overweight or obesity.
- HAZ: SDS of <−2 for stunting, SDS of –2 to +2 for normal values, and SDS of >+2 for tall stature.
- BAZ: SDS of <−2 for thinness, SDS of –2 to +1 for normal values, SDS of +1 to +2 for overweight, and SDS of >+2 for obesity.

Blood pressure (BP) measurements in children will be taken by a certified physician using an appropriately sized cuff and an aneroid manometer. Three BP measurements will be obtained after the participant has rested for 5 minutes in a seated position with their feet on the floor. The mean systolic and diastolic BP for each participant is calculated from the recorded readings. Heart rate is determined through palpation of the radial artery.

Parents will be interviewed to identify medico-social factors (eg, age, parental education, and income).

The variables assessed in this study are presented in Tables 2 and 3.

**Table 2:** Anthropometric and sociodemographic characteristics of the children and their parents.

Variable	Description
Age (y)	Quantitative
Sex	Female Male Intersex
Ethnicity	Kazakh Other Asian Russian Other European Refuse to answer
Region of residence	Aktobe Mangystau Atyrau West Kazakhstan
Place of residence	Urban Rural
Body length (cm)	Quantitative
Body weight (kg)	Quantitative
BMI (kg/m^2^)	Quantitative
Head circumference (cm)	Quantitative
Chest circumference (cm) at rest (normal calm breathing), at maximum inhalation, and at full exhalation	Quantitative
WAZ^a^	Quantitative
HAZ^b^	Quantitative
BAZ^c^	Quantitative
Mother’s age at the time of childbirth (y)	Quantitative
Father’s age at the time of the child’s birth (y)	Quantitative
Pregnancy sequence number	Ordinal
Baby’s birth weight (kg)	Quantitative
Child’s height at birth (cm)	Quantitative
Breastfeeding period (mo)	Quantitative
Physical activity	Low Medium High
Physical education and sports outside of school	Dormant Irregularly Regularly
Duration of child’s sleep (h)	Quantitative
Systolic blood pressure (mm Hg)	Quantitative
Diastolic blood pressure (mm Hg)	Quantitative
Resting heart rate (beats per minute)	Quantitative
Mother’s height (cm)	Quantitative
Mother’s weight (kg)	Quantitative
Father’s height (cm)	Quantitative
Father’s weight (kg)	Quantitative
Educational level of the mother	Higher Secondary specialized Secondary
Educational level of the father	Higher Secondary specialized Secondary
Family	Complete Incomplete
Number of children in the family	Quantitative
Living space per family member (m^2^)	Quantitative
Harmful and hazardous working conditions of parents	Yes No Refuse to answer
Parents working in the oil and gas industry	Yes No
Presence of pathology in the mother during pregnancy (if “yes,” specify which pathology)	Yes No
Smoking by the mother or father	Yes No Refuse to answer
Alcohol use by the mother or father	Yes No Refuse to answer
Income	Up to KZT 50,000 (US $92.35) KZT 50,000-100,000 (US $92.35-$184.70) KZT 100,000-150,000 (US $184.70-$277.06) KZT 150,000-200,000 (US $277.06-$369.41) KZT ≥200,000 (US $369.41)
Water consumption	Tap Bottled Borehole or well Filtered Tap+bottled water
Parents’ assessment of their child’s health	Excellent Good Satisfactory Bad Refuse to answer

^a^WAZ: weight for age *z* score.

^b^HAZ: height for age *z* score.

^c^BAZ: BMI for age *z* score.

**Table 3:** Chemical element content in the hair of school-aged children.

Element type	Elements	Category
Macroelements	Calcium, potassium, sodium, magnesium, and phosphorus	Quantitative
Essential trace elements	Chromium, cobalt, copper, iron, iodine, manganese, selenium, and zinc	Quantitative
Toxic trace elements	Aluminum, arsenic, beryllium, cadmium, mercury, and lead	Quantitative
Potentially toxic trace elements	Boron, lithium, nickel, silicon, tin, and vanadium	Quantitative

### Statistical Methods

Statistical processing of the results will be carried out using the SPSS (version 25; IBM Corp) and STATISTICA 10 (StatSoft) programs. The Kolmogorov-Smirnov criterion will be used to determine the normality of the distribution of quantitative variables. Variables with normal distribution will be presented as means and SDs, and variables with non-Gaussian distribution will be presented as medians and IQRs. Qualitative variables will be presented as absolute values and percentages. To assess statistically significant differences between quantitative variables, we will use the Student criterion, the nonparametric Mann-Whitney *U* test for 2 samples, and the Kruskal-Wallis *H* criterion for 3 or more samples. The Pearson chi-square criterion will be applied to identify intergroup differences for categorical variables. Multiple linear regression analysis will be applied to assess the association between chemical element content in hair and *z* score measures of children’s physical growth, as well as the association with gender, age, and residential status. Logarithmic transformation will be conducted for variables with non-Gaussian distribution. The critical level of significance for testing statistical hypotheses is assumed to be.05.

### Ethical Considerations

This research was approved by the institutional review board of the West Kazakhstan Marat Ospanov Medical University (meeting number 7.02, dated September 29, 2022). Written informed consent was obtained from the legal guardians of the participants. Participation was voluntary, and the participants had the right to withdraw from the study at any time. The original informed consent form or institutional review board approval cover secondary analyses using existing data with primary consent without the need for additional consent. The data are anonymized. The participants are identified by a numerical code. No compensation is provided to participants for this research.

### Data Reporting and Visualization

From the data obtained, a web-based mapping will be carried out by color according to the concentration of chemical elements in children’s hair. The data will be presented as a web-based atlas. The mapping is conducted using the cross-platform geographic information system software QGIS (version 3.18; QGIS Development Team). Vector data are taken from public sources. The color in the map represents the median chemical element content in hair

The atlas is being developed as a web application using modern adaptive design, where the site layout will change automatically depending on the user’s screen. Data entry will be limited to authorized users, and access will be granted by the web application administrator.

## Results

Recruitment of participants and data collection will occur between September 2023 and March 2025. Data processing and analysis will be completed in September 2025. Publication of the results is expected in December 2025. Table 4 shows an outline of the study plan.

**Figure 2:**
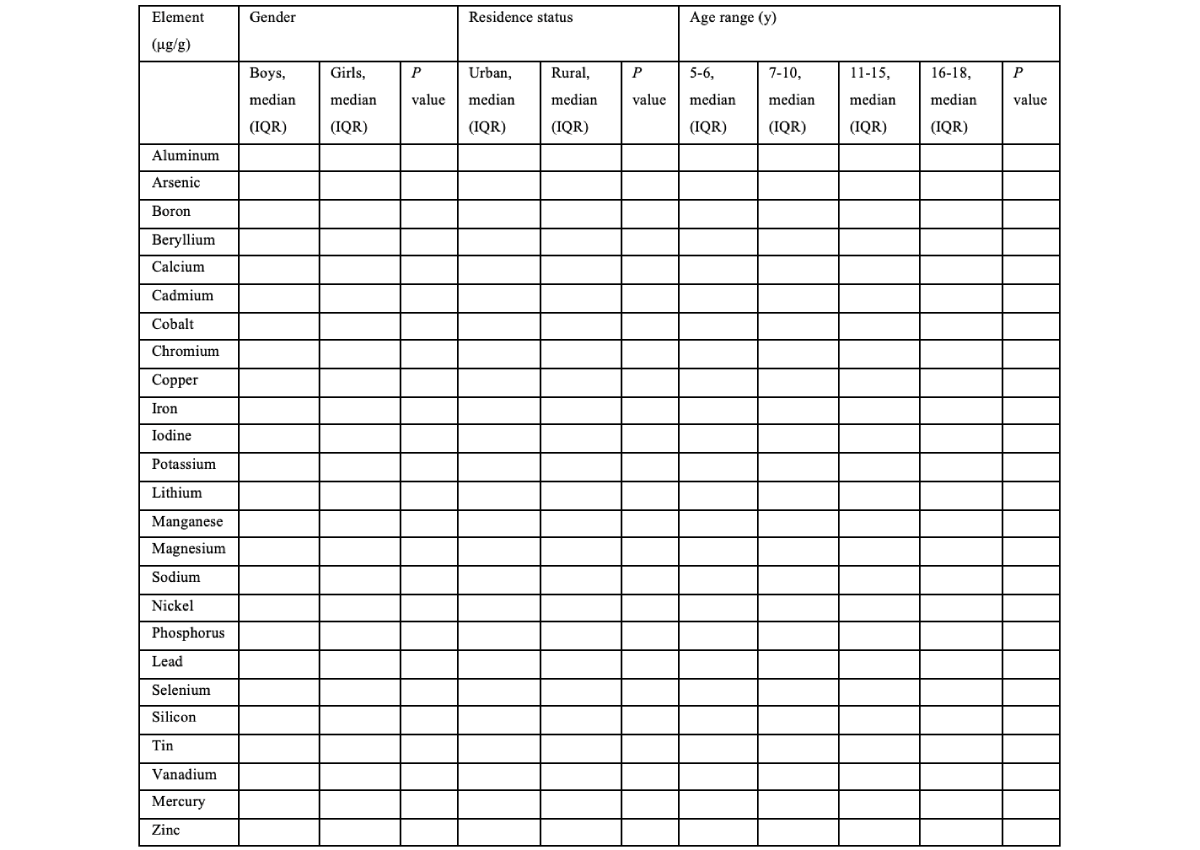
Sample table of the comparative analysis of the chemical element content in children’s hair.

Data on the trace element content in children’s hair will be obtained (Figure 2). The chemical element content will be reflected in the web-based atlas, entitled “Elemental status of the child population of the Western region of the Republic of Kazakhstan.” The electronic atlas containing 25 maps (aluminum, arsenic, boron, beryllium, calcium, cadmium, cobalt, chromium, copper, iron, iodine, potassium, lithium, manganese, magnesium, sodium, nickel, phosphorus, lead, selenium, silicon, tin, vanadium, mercury, and zinc) of the elemental profile of the child population of the western region of Kazakhstan will be available on the website [66]. Reference values for the 25 chemical elements in hair will be obtained. An analysis will be conducted to determine the differences in the levels of elements in groups of boys and girls, urban and rural children, and groups of children of different ages. According to the results of multiple regression analysis, chemical elements influencing the indicators of physical development of children will be identified.

The prevalence of physical development and growth disorders according to the WHO 2007 criteria will be determined. The incidence of normal and abnormal physical growth of children and adolescents by gender and residence status will be presented.

**Table 4:** Quarterly scheduling of research work.

Item	2023					2024					2025			
	Quarter 1	Quarter 2	Quarter 3	Quarter 4	Quarter 5		Quarter 6	Quarter 7	Quarter 8	Quarter 9		Quarter 10	Quarter 11	Quarter 12
Outline of study design and preparation	✓													
Reference collection and review of the literature	✓	✓												
Data collection			✓	✓	✓		✓	✓	✓	✓				
Data compilation and tabulation								✓	✓	✓		✓		
Data analysis										✓		✓	✓	
Thesis writing										✓		✓	✓	
Final thesis preparation												✓	✓	
Submission														✓

## Discussion

### Expected Findings

To assess the elemental profile of children, we will apply polyelemental analysis of hair. In biomonitoring studies, hair is a reliable biomarker for assessing the elemental profile of children and adolescents, as well as for analyzing the impact of environmental pollutants [67]. Elemental analysis of hair allows not only for the assessment of the current status of the human body but also for the reconstruction of past episodes of exposure and nutrition even if the action has already ceased. The growth rate of human hair is approximately 10 mm per month, and the level of elements in hair reflects their levels in the body over time, whereas the concentration of metals and metalloids in blood and urine changes rapidly after exposure (within a few days for blood and a few weeks for urine). Hair analysis has several advantages [46]. Hair is easy to take, collect, store, and transport, and the collection procedure is noninvasive. Chemical elements accumulate in hair at higher concentrations and persist longer than in blood, urine, and saliva [68].

Although the applicability of hair analysis is debated, the standardization of sample collection, reporting procedures, and analytical methods, as well as quality management, allow hair to be used as an effective marker in epidemiological studies. A clear methodology for such studies is ICP-MS, which can detect nanograms of several components of scalp hair and which we will use in this study [67,68].

There are many studies in scientific literature using elemental analysis of hair from children and adolescents to assess the impact of polluting factors in different countries, including Russia [69], Bolivia [38], Italy [23,24], Ethiopia [17], Japan [31], and Colombia [39]. It has been found that children living in polluted areas near industrial enterprises have significantly higher heavy metal and lower essential trace element content in bioassays. It is assumed that the adverse health effects on children and adolescents living near factories and industrial plants may be related not only to the excess of toxic metals but also to changes in mineral homeostasis. However, it should be recognized that the number of studies determining the concentration of chemical elements in hair in populations of children living in polluted areas is lower than that of similar studies conducted in adult populations. In addition, a significant part of contaminated territories and exposed populations, including children, has not been covered by such studies at all.

It should be noted that environmental pollution is not the only source of toxic metals in children’s bodies. When conducting biomonitoring studies of children, the intake of trace elements (including toxic ones) from food and water should be considered [12,17,31]. Other important considerations include harmful habits such as smoking among family members [30,52,53], social status and income, the level of education of the parents [31,41,52,70], and ethnicity. Furthermore, the child population comprises different age groups, which differ not only in anthropometric data but also in the level and intensity of physiological processes associated with various stages of growth and development. There are also differences in social activity and the formation of behavioral patterns. This makes younger children more vulnerable to toxic trace elements than older children [13-16]. The relationship between chemical elements in hair and children’s health is most often assessed in case-control studies involving groups of healthy children and children with diseases such as anemia [56]; night blindness [57]; myopia [71]; amblyopia [72]; inflammatory bowel disease [73]; autism spectrum disorder [74-78]; behavioral disorders [79]; Down syndrome, obesity, and growth delay [80]; attention-deficit/hyperactivity disorder [81-83]; cerebral palsy [84]; and developmental language disorder [85]. Some studies consider anthropometric [17,70,86], biochemical [86], and physiological [86] indicators as characteristics of children’s health.

Hair is a useful tool in assessing the supply of zinc [87,88], selenium [89], copper [90], calcium, and magnesium to the child population [61].

Our project is a continuation of our previous cross-sectional study on the assessment of elemental profile conducted in adults aged 18 to 60 years. On the basis of the analysis of microelement status, medical and biological monitoring of population health at the population level was carried out [91,92]. The obtained data indicated that, among residents of western Kazakhstan, living near oil and gas fields affected the copper, iodine, aluminum, mercury, and lead content in hair. The increasing aluminum and mercury content was associated with decreasing distance to oil and gas fields, and the increasing copper, iodine, and lead content was associated with increasing distance to these fields. Our results demonstrate the need for regular biomonitoring of chemical elements to determine long-term temporal trends in the impact of chemicals on the health of the population of western Kazakhstan [58,59]. In the absence of state programs for biomonitoring the element content in the bodies of the population, which involve studying the population over a long period of time , the temporal trends of exposure to chemical elements can also be assessed taking into account the ontogenetic approach (ie, including different groups of the child population in a cross-sectional study).

The reference values of trace and macroelements obtained as a result of our study are recommended to be used for biomonitoring and environmental risk assessment in Kazakhstan. Our team includes researchers with experience in assessing and presenting the elemental profile of residents of the western region of Kazakhstan [58,59,93].

This study is conducted within the framework of the implementation of a scientific project with grant funding from the Committee of Science of the Ministry of Science and Higher Education of the Republic of Kazakhstan (“Elemental status of the child population of the Western region of the Republic of Kazakhstan”; grant AP19677517) (Multimedia Appendix 1).

We assume that the presentation of the results of our study in the form of an atlas of elemental profile will facilitate the perception of our findings.

We welcome collaboration with other researchers; our data can be made available upon request. We provide an opportunity for researchers to post data on the elemental profile of the populations they study on our website, with the possibility of visualizing them in the form of maps.

The results and conclusions of our study can be compared with data from other studies investigating the elemental profile of populations.

### Limitations of This Study

The study design is limited to a single-stage cross-sectional study, with no opportunity to observe changes in elemental profile over time. We did not consider dietary intake of trace and macroelements. In addition, when interviewing mothers, their responses may be influenced by memory distortions.

### Conclusions

A child’s growth trajectory not only indicates their current health but also predicts long-term outcomes such as cognitive development, susceptibility to disease, and productivity in adulthood. Given the vulnerability of children and the region’s unique geochemical and industrial characteristics, assessing physical growth alongside elemental profile provides a comprehensive picture of children’s health and potential environmental exposure. Understanding the relationship between elemental profile and growth indicators can reveal the impact of environmental exposure on children’s health in the region. Such findings could inform the development of region-specific public health measures such as nutritional supplements, environmental remediation, and policies aimed at reducing exposure to toxic elements. In addition, identifying differences between rural and urban populations could inform the development of targeted prevention strategies. The developed web-based atlas of trace and macroelement content will be necessary for further research on the prevalence, etiology, risk factors, and possible mechanisms of development of environmentally dependent, endemic diseases in the region.

## Appendix

Multimedia Appendix 1 Peer-review report by the Committee of Science of the Ministry of Higher Education and Science of the Republic of Kazakhstan, National Centre for Scientific Technical Expertise.

## Abbreviations

BAZ: body mass index for age *z* score
BP: blood pressure
HAZ: height for age *z* score
ICP-MS: inductively coupled plasma mass spectrometry
SDS: standard deviation score
WAZ: weight for age *z* score
WHO: World Health Organization

## Data Availability

The datasets generated or analyzed during this study are available from the corresponding author on reasonable request.
