# Supplementary material for: Association Between Hair Trace Element Content and Children’s Growth and Development: Protocol for a Cross-Sectional Surveillance Study
Source: JMIR Res Protoc. 2025 Sep 16;14:e72207. doi: 10.2196/72207 (PMC12485260; doi:10.2196/72207)
Supplement: Multimedia Appendix 1 [file resprot_v14i1e72207_app1.pdf]

(/)

Notification 0

Батырова Гульнара Арыстангалиевна

(https://is.ncste.kz/announce) Announcements 0

Back

AP19677517

IRN: AP19677517 Elemental status of the child population of the Western region of the Republic of Kazakhstan

Chat with us

№	Name of assessment criterion	Rating (from 0 to 9)	All reviewer comments with answers to leading questions		
1	The novelty, relevance and viability of the project	6.33	The project aims to examine the level of elements in a large number of children in Western Kazakhstan. The study is cross-sectional and aiming at a large enough sample. The project aims were clear, and also it was easy to visualise what exactly would be delivered. This project, if well conducted, is likely to be of international interest, and the sample size is large enough to guarantee that (assuming the applicants can deliver it). The importance of the project was also made clear in the application. The background section was very good, and appropriately referenced. The aims and methods section were well structured and clear. The methods section was excellent, with the aims linked to deliverables and clear methods and sampling targets. The literature and references were very good, and it was made clear why this work is needed.	The proposed programme is highly relevant mainly from national point of view, since children living in some parts of West Kazakhstan may be exposed to toxic elements, mainly heavy metals, due to the heavy industry situated in this region. The proposed project is innovative from national point of view, since many developed countries have been usually monitoring the stage of the pollution in highly exposed areas and its effect on the health of local populations. The literature referred to by the authors of the project is relevant and actual.	The applicants propose to study the state of the elemental status of the child population as an indicator of the availability of micronutrients for children and the impact of environmental pollutants, especially in rural areas. The argue that this will, for the first time in Kazakhstan, allow the influence of elemental status on physical development to be assessed using WHO standards. The novelty of the work therefore comes out of the collection of this new dataset. The applicants do not propose scientific hypotheses and the project is not attempting to answer a research question but is rather attempting to undertake population surveillance to estimate the magnitude of the problem of nutrient deficiency in the population. The approach taken is appropriate. The literature is well referenced.

№	Name of assessment criterion	Rating (from 0 to 9)	All reviewer comments with answers to leading questions		
			The problem the study aims to address is important, although it is purely epidemiological work. However, epidemiological work is needed and is always the first step as is the case here. There were quite a few methodological details, even on statistical analyses. It is expected the findings will be of interest to international journals. The project has significant elements of novelty. The project is reasonably aligned to global research trends in this research space, with children's health a global priority. The specific aims were clearly described, as was for the overarching aim and the need for the project. Target journals were not mentioned by name, but they meet the requirements of the call documentation. The authors could have clarified what exactly will be pursued in each of the articles they promise, but there is capacity for many outputs in this project.	The proposed project reflects current trends in the field. The example of the following of global trends is the usage of WHO standards. The influence of elemental status on physical development of children will be assessed using WHO standards. The data resulting from the proposed project have a high potential to be published in international peer-reviewed journals. The publication plan is adequate. At least three papers are planned to be published in international peer-reviewed journals.	From the point of the impact on population health, the applicants ague that the work will provide required information to allow any mico- and macro elements of the nutrient profile of children to be corrected via dietary methods. As such the work has potential applied impacts although there are challenges in translating results recorded in a sample of children with individually tailored therapeutic interventions (i.e. just because there might be general deficiency in some micro nutrients in an area, it's not the case that supplementation would be appropriate for all children). In terms of global trends this sort of approach (mapping the scale of the problem) is perhaps behind many other countries where similar publications have already been produced, although the development of an on-line interactive atlas is one novelty of this proposed project. Because of the general lack of originality though, coupled with the fact that it is unclear the degree by which findings might be of interest to scientists outside Kazakhstan, this work might be challenging to publish in the international peer-reviewed literature. The article expectation is

№	Name of assessment criterion	Rating (from 0 to 9)	All reviewer comments with answers to leading questions
			likely to be an over-estimate of the true number as this is not a project that will lead to breakthrough science (although it is one that may be of local benefit).

№	Name of assessment criterion	Rating (from 0 to 9)	All reviewer comments with answers to leading questions		
2	Quality and feasibility of the research plan	7	The research plan was very good, with only a couple of limitations. The sample is large enough, which is major plus. The research problem and the need for this work were made clear. The aims were specific and also clear and linked to deliverables in the methods section.	The aims, objectives and hypotheses of the research plan are clearly stated and realistic.	The applicants argue that the research the undertake will directly address the national project "Quality and affordable healthcare for every citizen" Healthy Nation ", where special attention is paid to preserving and strengthening the health of children. They show how Western Kazakhstan is a developed industrial region with the oil and gas industry, electric power industry, ferrous and non-ferrous metallurgy, fuel, chemical and petrochemical industries, building materials industry, which are characterized by high resource intensity and the production of significant amounts of waste. This it is suggested could influence the elemental characteristics of the body composition of children living within the region. They therefore aim to undertake a survey of just over 1000 children, use hair samples to measure elemental composition, combine with anthropometric measures and map using an online atlas. The broad approach is clearly described although it is not supported by scientific hypotheses.

№	Name of assessment criterion	Rating (from 0 to 9)	All reviewer comments with answers to leading questions		
			The research plan was very good and clear. I particularly liked the risk mitigation section. Other aspects that were good included the large sample, the clarity on the outcomes, the methodological description and the software to be used. The use of a website to present results is also a positive. The two main issues of concern were: 1) the decision on the sample was not driven by power calculations; 2) the sampling strategy was not clear, how it would ensure a good spread of recruitment through the region of interest. The practical aspects of the sampling could have been clarified a bit more.	Research methodology is well described. A non-invasive approach to study toxic element load in children was selected, which is welcome. Multielement hair analysis will be performed using coupled plasma mass spectrometry in sufficient amount of children (n=1094). However, it is not clear, why reference values for the content of chemical elements in biological substrates of the population will be assessed. It is not clear, why reference values already used in developed countries will not be used as standard cut-offs. The ethical issues and the issues relating to the prevention of plagiarism, falsification, and fabrication of data, false co-authorship, and the assignment of results are properly discussed.	The broad method proposed (a population-based sample of children) is appropriate. However, there are a number of uncertainties. 1) The applicants propose to study 1094 children, but it is not clear where this number came from and hence it is unclear how appropriate the sample size will be for the needs of the research. 2) The applicants state that they will use “cluster sampling” but no information is given on this. They do not describe what the clusters will be and provide no information on how children will be recruited to the study. Many of the participants will be below the age of consent and therefore recruitment will need to be via parents, but it is not clear how this will work. 3) The applicants propose to collect information on body weight and status but the need for this was not clear. It seems like a potentially unnecessary addition to the work, the aim of which is to map elemental status. The measures add to the intrusion and cost of the work. Given there is already evidence on the relationship between weight status and elemental composition, this may not be needed. 4) The applicants propose to produce an interactive atlas but the

№	Name of assessment criterion	Rating (from 0 to 9)	All reviewer comments with answers to leading questions
			geographical units via which data will be mapped are not detailed. This is important because the population size of each unit will influence the uncertainty around mapped values and given the total sample is relatively small, then it is likely that quite large geographical units will be required which would make the maps less useful. A related concern links to sampling; how do the applicants propose to ensure an adequate number of children are sampled in each unit? The applicants have addressed issues of plagiarism and falsification adequately and discuss the use of reference cards. The appear to be following appropriate ethical procedures.

№	Name of assessment criterion	Rating (from 0 to 9)	All reviewer comments with answers to leading questions		
			The aims of the project were clear, as were the deliverables. Aims and deliverables were clearly linked in the very good methods section. The expectation would be for the project to meet all of its aims, assuming recruitment goes to plan. Specific target journals were not provided. The project can publish in an international journal and the findings will be of interest to large number of international readers. Risk for the successful completion of the study include: 1) recruitment challenges 2) poor data recording and management.	There is a very high probability to achieve the expected results.Some risks of the successful completion of the study (such as the possibility of novel pandemy of COVID-19 and refusal of parents to participate in the study) are mentioned and the mitigation plan is proposed. There is a potential to publish the data in international peer-reviewed journals.	The broad approach adopted appears to be achievable in the time available, although the lack of detail on the sampling methodology the applicants propose is a concern. It is likely that results might be accepted for publication in Kazhakstan but the lack of generalisable scientific hypotheses might mean that there is less interest in the international scientific community. The main risk of completion is the inability to sample the required number of children. The applicants have considered this (as well as the potential impacts of Covid-19). They state that in cases of recruitment problems they will hold explanatory conversations with parents, posting promotional information in the media, social networks about the non-invasiveness and potential usefulness of the planned study, involving doctors from regional healthcare institutions. These are appropriate actions although, as detailed, their utility would be strengthened by more detail on proposed recruitment. In terms of achievability, it appears that the applicants propose to complete recruitment of children by the end of 2023 which is a very ambitious timescale. In

№	Name of assessment criterion	Rating (from 0 to 9)	All reviewer comments with answers to leading questions
			2024 they state that they will “continue studying the content of chemical elements in biological substrates with a trip to the settlements of Atyrau and Mangystau regions” and in 2025 they will undertake “a study of the content of chemical elements in biological substrates with a visit to the settlements of the West Kazakhstan region”. These “biological substrates” studies are somewhat confusing as it is not clear how they fit with the primary research need (measure elemental status in children). This may be a problem with translation of the application to the English language as the activities table provided at the end of the application suggests that hair samples will continue to be collected in 2024 and 2025 but this was unclear.

№	Name of assessment criterion	Rating (from 0 to 9)	All reviewer comments with answers to leading questions		
3	Expected results and their significance	6.67	The total was 99068 thousand tenge, which is relatively low for the sample promised. The tasks and subtasks were well detailed. The authors could have provided a Gannt chart.	The budget for personnel (team members), purchase of materials/consumables (each item is listed), scientific and organizational support (publication costs, registration costs for the conferences, laboratory services), and business trips is requested. The purchase of novel general equipment (monitor, PC) is planned. The requested items are fully justified.	Broadly this is appropriate although the potential efficiency of the project is somewhat challenging to investigate due to the limited amount of information provided on sampling and recruitment. In terms of potential efficiency gains it appears that the measures of anthropometry in the children could potentially not be required without reducing the scientific validity of the work

№	Name of assessment criterion	Rating (from 0 to 9)	All reviewer comments with answers to leading questions		
			The study can certainly be featured alongside other international developments in this research space. There are a lot of novel aspects in the research question, but even if there were not the research question is still important to the target area in Kazakhstan – it should be a public health priority. Thus, it would be expected that the findings would be certainly relevant to Kazakhstan. National impact is certain, with international impact very likely. There is work in this research space, but in a different setting. Therefore, there is a need for this project. If well conducted, it will be a useful addition to the international literature in this research space. The project may be able to deliver a high impact factor publication. Finally, the significance of the expected results, and the impact, was well explained.	The expected results may be applied in the area of preventive toxicology, medical geography, epidemiology, and paediatrics. To strengthen the socioeconomic impact of the proposed project, a close cooperation with national bodies is required to implement corrective and preventive measurements. The cooperation with national bodies is just indicated as a possibility in the proposed project and not a key part of the project. There is a potential to publish the data in international peer-reviewed journals. The involvement of junior researchers under the age of 40 years in the scientific team is planned.	The work could potentially be used to develop remediation measures for either elemental deficiencies (via diet) or excesses (via exposure to air, water, or soil pollution). The applicants do not detail what the nature of these interventions might be however, and there is no clear pathway described in the application that would lead to them. The approach adopted (population surveillance via a survey of children) is an appropriate one. The atlas will have intellectual property associated with it, but it is not clear that this work will lead to the production of outputs that are suitable for commercialisation. As stated earlier in this review, articles produced are likely to be of interest to scientists in Kazakhstan, but the international interest is less clear. The specific involvement of young scientists was not addressed although it appears two of the team will use the project to obtain doctoral theses.

№	Name of assessment criterion	Rating (from 0 to 9)	All reviewer comments with answers to leading questions		
4	Competence and scientific groundwork of the research group	7.33	The primary investigator’s publication record is good, but more information could have been provided on their publication metrics, like the total number of papers and citations. Information like that could serve as an indication of the quality of the delivered previous work. However, the primary investigator has experience in leading previous projects, which is a major plus.	The primary investigator is a highly experienced general practitioner. The primary investigator led several scientific and technical projects. Unfortunately, international publication record of a primary investigator is limited (h index 3).	The project manager has an H-index of 3 which suggests they do not regularly publish in international peer reviewed journals, but they do have some papers in the international literature. They have a particular focus on topical problems of medical elementology and the prevalence of element deficiency in the child and adult population. They have undertaken previous work to study factors affecting the content of elements in the population of Western Kazakhstan and although this is mentioned a various points in the application the way by which this new project would build on (rather than duplicate) existing work is unclear. It appears perhaps this previous work was undertaken in adults rather than children. It appears that they have previous experience of leading projects of a similar scale to this.

№	Name of assessment criterion	Rating (from 0 to 9)	All reviewer comments with answers to leading questions		
			The team is large with some very experienced researchers, which is an advantage. In addition, the relevant table listing the background and what each member will bring to the project (Table 1) has been completed very well, with a lot of information on the relevant expertise of the applicants and their roles in the project. From that table it was clear that the team is also highly multidisciplinary, another major plus. The downside is that young researchers do not appear to have been included, at least as co-applicants. The same stands for international collaborator, it is lacking, and that could have boosted the project greatly.	The research team is composed of several senior and junior researchers with appropriate qualifications in ecology, endocrinology, paediatrics, family medicine. International publication record of some senior team members is good, in others it is still limited (h indexes are ranging from 1 to 7). The role of each team member in the proposed project is clearly stated. The participation of foreign scientists from Russia to perform hair sample analysis is planned. The purchase of novel general equipment (monitor, PC) is planned.	The research teams are broadly appropriate given the scale and nature of the project. The roles are clearly described. There are three members of the team who have an h-index score of 4 or above. The qualifications of the team members are matched well to the project. Other than office consumable the equipment purchases will be to allow analysis to be undertaken of the elemental composition of hair samples. It appears that the team members are suitably qualified to use this equipment and compete analysis. It does not appear that any foreign scientists are proposed to be involved in the project.

№	Name of assessment criterion	Rating (from 0 to 9)	All reviewer comments with answers to leading questions		
			The infrastructure is appropriate. Third party contractors will not be used. The equipment needed within the project seems appropriate. The project will definitely improve the standing of research in Kazakhstan.	The appropriate infrastructure is at disposal. All facilities and the equipment needed to carry the research work are in place. The purchase of novel general equipment (monitor, PC) is planned.	The research infrastructure is appropriately matched to the needs of the project and indeed the research team have a history of having completed similar work to that being proposed here. The applicants state that hair samples for analysis are planned to be sent to the laboratory of OOO Molecular Medicine (Moscow). Multielement hair analysis will be performed there using inductively coupled plasma mass spectrometry (ICP MS) using an Agilent 8900 (USA) mass spectrometric detector with a triple quadrupole. The applicants state that the determination of the content of chemical elements in hair using this method makes it possible to comprehensively assess the impact of environmental and hygienic factors on the human body.

№	Name of assessment criterion	Rating (from 0 to 9)	All reviewer comments with answers to leading questions		
5	Multidisciplinarity of the project	2	The project has many multidisciplinary elements, for example including spatial epidemiology, biological measurements, children's health and statistical analyses. The project was of high enough quality to give confidence in its multidisciplinary deliverables.	The proposed project is multidisciplinary.	The project is interdisciplinary in terms of ensuring cooperation between broad scientific fields, an interdisciplinary approach is fully justified in the application and is necessary to achieve the goal of the project. This is because the project team includes scientists with expertise in medical science, environmental chemistry data science, and data visualisation.
Final score		29.33	Total points for all of the evaluation criteria above		
Justification of requested funding			The funding requested is acceptable, and perhaps even low considering the sample size.	The budget for personnel (team members), purchase of materials/consumables (each item is listed), scientific and organizational support (publication costs, registration costs for the conferences, laboratory services), and business trips is requested. The purchase of novel general equipment (monitor, PC) is planned. The requested items are fully justified.	The funding is appropriately matched to the requirements of the project. No changes are suggested although it is possible that the applicants could remove the component of the project that will be making anthropometric measurements if there was a need to save money as these are costly and the scientific justification for them was not clear.

№	Name of assessment criterion	Rating (from 0 to 9)	All reviewer comments with answers to leading questions		
	Compliance with priority		The application is relevant to the priority area (The Science of Life and Health) and to the specialised scientific area (Innovative research in medicine and public health), under which it was submitted.	The proposed project is categorized within the priority area Life and Health Science, field of research: Innovative research in medicine and public health, type of research: applied. This categorization fully corresponds to the project goals.	The project is entitled “Elemental status of the child population of the Western region of the Republic of Kazakhstan". This fits well with Priority area 6. “The science of life and health” and sub-priority (specialised scientific direction) area 6.3 “Innovative research in medicine and public health”.
	Strong points		Important area of research. Multidisciplinary project. Good principal investigator. Large enough, experienced and multidisciplinary team. Good introduction and references. Very good methods section. Clear aims and deliverables. Large sample.	The proposed programme is highly relevant from national point of view, since children living in some parts of West Kazakhstan may be exposed to toxic elements, mainly heavy metals, due to the heavy industry situated in this region.	- The application addresses an important public health issue for children’s health, a national priority. - The approach uses appropriate techniques. - The application is from a team with previous experience in this field, enhancing the chances of success.
	Weaknesses		Not clear how the sample size was determined. The practical aspects of the sampling could have been explained better. Young researchers do not appear to be involved as applicants. International collaborator involved.	It is not clear, why reference values already used in developed countries will not be used as standard cut-offs. The cooperation with national bodies is just indicated as a possibility in the proposed project and not a key part of the project.	- The sampling method that will be used was not described clearly and justification for the target sample size was not provided. - The need for the measurement of anthropometric characteristics of children was not made clearly. - Activities to be undertaken in years 2 and 3 of the project were not clearly described.
